# Three-dimensional motions of distal syndesmosis during walking

**DOI:** 10.1186/s13018-015-0306-5

**Published:** 2015-10-24

**Authors:** Chen Wang, Junsheng Yang, Shaobai Wang, Xin Ma, Xu Wang, Jiazhang Huang, Chao Zhang, Li Chen, Jian Xu, Xiang Geng, Kan Wang

**Affiliations:** Department of Orthopedics, Huashan Hospital, Fudan University, NO.12 Middle Wulumuqi Road, Jingan District, Shanghai, China; Harvard Medical School, Boston, MA USA; Key Laboratory of Exercise and Health Science, Ministry of Education, Shanghai University of Sport, Shanghai, China; Department of Radiology, Huashan Hospital, Fudan University, Shanghai, China

**Keywords:** Syndesmosis, Six DOF, In vivo kinematics, 3D to 2D registration

## Abstract

**Introduction:**

The motion of the distal syndesmosis correlates highly with the instability, while an accurate kinematic description of the distal tibiofibular joint during normal gait has not previously been presented.

**Material and methods:**

Sixteen healthy syndesmoses of sixteen living subjects (8 male and 8 female) were studied during stance phase of the normal gait. Data of CT scanning were collected first and used to create the 3D models of the distal tibia and fibula. The lateral X-ray images of the syndesmosis were captured by fluoroscopy when the subject was walking. Seven key-pose images were selected for subsequent 3D to 2D bone model registration and six degrees-of-freedom (DOF) motions of syndesmosis were then calculated. A validation experiment was also conducted to confirm the accuracy of the 3D/2D technique for the syndesmosis.

**Results:**

During the stance phase, the distal tibiofibular joint exhibited with 2.98 ± 1.10° of dorsi/plantarflexion, 5.94 ± 1.52° of inversion/eversion, and 5.99 ± 2.00° of internal/external rotation; 2.63 ± 1.05 mm on medial/lateral, 3.86 ± 1.65 mm on anterior/posterior, and 4.12 ± 1.53 mm on superior/inferior translation. From heel strike to mid-stance, the syndesmosis demonstrated 1.69° of dorsiflexion, 3.61° of eversion, and 3.95° of external rotation. Likewise, from mid-stance to heel-off, the syndesmosis presented 1.04° of plantarflexion, 4.95° of inversion, and 5.13° of internal rotation.

**Conclusion:**

During the stance phase of normal gait, internal/external rotation and vertical motion play dominant roles in terms of rotation and translation, respectively.

## Introduction

The syndesmosis locates between the triangular fibular notch of the lateral surface of the distal tibia and the medial convex surface of the distal fibula. It is an important fibrous joint united by powerful interosseous ligament to resist forces that attempt to separate these two bones [1, 2]. Because of the asymmetric shape of the trochlea of talus and the elastic fixation of fibula to tibia, the movement between fibula and tibia is 3D and functionally coupled to the ankle joint. Damage to normal kinematics of the tibiofibular joint may cause recurrent ankle sprains and even chronic ankle instability [3]. However, the in vivo kinematic data of the distal tibiofibular joint is still limited compared with other joints such as shoulder and knee [4–6], which has impeded the improvement of clinical diagnosis, treatment, and evaluation of the syndesmosis after injuries.

In vitro cadaveric studies were chosen by some investigators to explore the rotational and translational motion of the distal tibiofibular joint in non weight-bearing condition, and some used dynamic cadaver-walking simulator in their investigations [7–9]. However, both have shown some defects as they cannot completely reproduce the real gait motion and reflect physiologic data in the normal.

In vivo kinematic data collected by surface markers or intraosseous pins attached to living subjects also had some flaws [10–13]. Surface markers cannot avoid relative movement between skin and the bone, and the intraosseous pins can cause invasive trauma, restricted motion, and relative severe ethical arguments.

The fluoro-based technique has been proved in previous kinematic researches to be effective to measure the in vivo kinematics of the bones and joints [4, 5, 14–19]. Although it is also not free from ethical concerns and may have constraint on tested motion speed, such technique was non-traumatic compared with intraosseous pins and much more accurate than surface markers.

As the 3D/2D registration technique had shown to be effective and accurate to obtain in vivo movement data in human joints such as shoulder, hip, knee, and ankle joint [4, 10, 14, 15, 18–20], The current study aims to investigate the in vivo six degrees-of-freedom (DOF) motions of the distal tibiofibular joint during stance phase of normal gait by 3D/2D registration technique.

## Materials and methods

### Study population

Sixteen healthy subjects between 24 and 46 years of age (8 male and 8 female) were recruited for this study. The mean age of the subjects was 37.5 ± 6.2 years old, with the average height of 1.68 m, weight of 68.3 kg, and body mass index (BMI) of 24.13. All subjects confirmed no obvious ankle sprains and traumatic or surgical history on both sides of the lower limbs. Before CT scanning, a qualified foot and ankle surgeon was asked to rule out any deformities or loss of joint motion in both ankles of each subject, and the gait patterns of the subjects were also confirmed to be normal. The consents are explained to each subject, and each subject has signed the experimental agreement. The study is approved by Huashan Hospital ethics committee (ethics statement number: HIRB 2015-037).

### CT imaging and 3D reconstruction

CT scanning of the tested lower leg was conducted for each subject after physical examination. The scanning scale ranged from 10 cm above the ankle joint to the bottom of the heel. Main CT scanning parameters included the following: thickness, 0.67 mm; voltage, 120 kV; current, 200 mA; and image matrix, 512 × 512 pixels. The outlines of the tibia and fibula bones were identified on all thin-slice CT images by density difference between bones and soft tissue. The 3D models of these bones were then reconstructed by the 3D reconstruction software (Amira 5.3.2, Visage Imaging, Inc., Berlin).

**Fig. 1:**
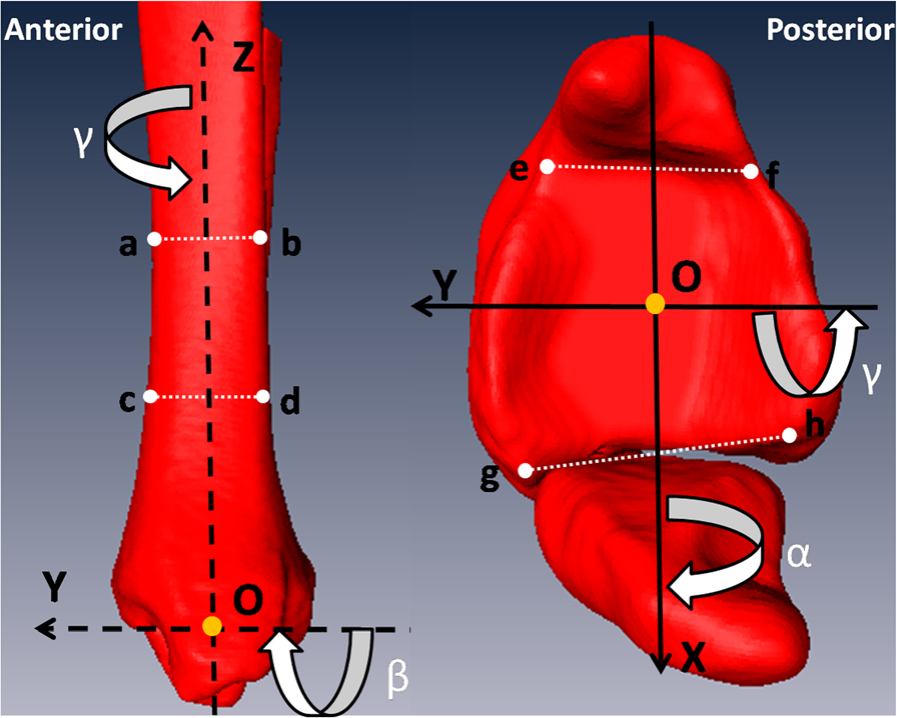
Medial view and inferior view of the syndesmosis. The line connecting the two mid-points of the distal tibial shaft at 5 cm (points *c*, *d*) and 10 cm (points *a*, *b*) above the joint surface was defined as the Z axis (from inferior to superior). The points of *e* and *g* represent the most medial and lateral points of the anterior tibial edge while points *f* and *h* represent the most medial and lateral points of posterior tibial edge. The X axis (from medial to lateral) was determined by connecting the midpoints of lines ef and gh. The Y axis (from posterior to anterior) was a cross product of the X and Z axes. The origin (point *o*) was the midpoint of the line which connecting the midpoints of lines ef and gh. The positive or negative value of the motion surrounding a specific axis was based on the right-hand rule. *α* presents for dorsiflexion(+), *β* presents for inversion(+), and *γ* presents for internal rotation(+)

The anatomical coordinate systems were constructed in both bones following the method of Yamaguchi et al. [18] using a custom software (Geomagic Studio 12, Geomagic, Research Triangle Park, NC). The directions of the XYZ axes and the positive-negative value of the motion surrounding a specific axis were all based on the right-hand rule (Fig. 1).

### Syndesmosis configuration in seven key-poses

Before the tests, a custom calibrator was placed on the receiver side of the fluoroscopy to adjust subsequent X-ray distortion when performing 3D/2D registration. During the tests, each subject walked for three continuous steps at a low speed of approximately 0.5 m/s to avoid abnormal gait. The subject’s syndesmosis was captured by a one-plane fluoroscopic device (BV Pulsera, Phillips Medical, USA) during the second step. The fluoroscopy was positioned on the horizontal plane to capture the lateral view of the syndesmosis at a rate of 30 Hz. Meanwhile, a high-speed camera started to take photos at a speed of 3000 Hz synchronously with the fluoroscopic device.

**Fig. 2:**
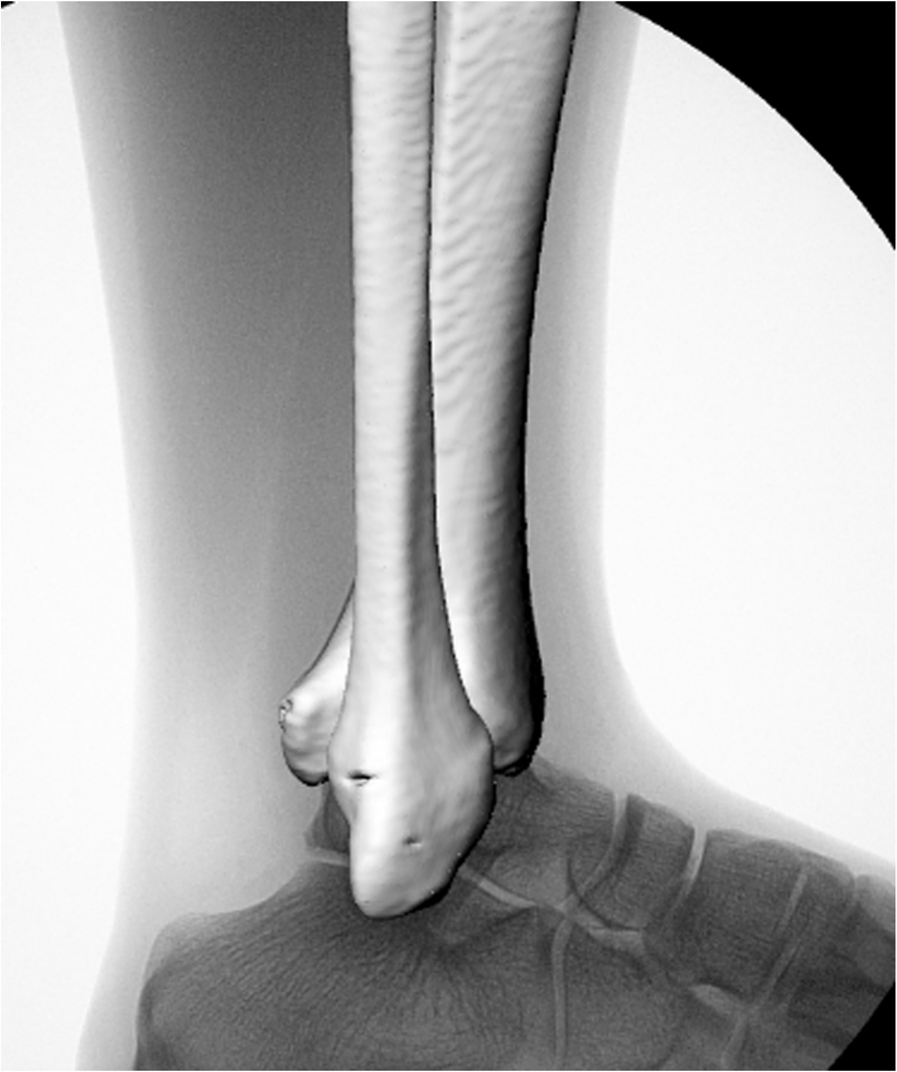
Seven key poses selected during one stance phase for 3D to 2D registration

Among the fluoroscopic images taken during a complete stance phase, seven key poses were found through high-speed camera photos: (1) preparation for heel strike of the tested side and heel-off of the contra-lateral side, (2) heel strike of the tested side, (3) mid-stance of the tested side and toe-off of the contra-lateral side, (4) mid-stance of the tested side and swing phase of the contra-lateral side, (5) heel-off of the tested side and preparation for heel strike of the contra-lateral side, (6) heel strike of the contra-lateral side, and (7) toe-off of the tested side and mid-stance of the contra-lateral side (Fig. 2).

### Six DOF evaluation

**Fig. 3:**
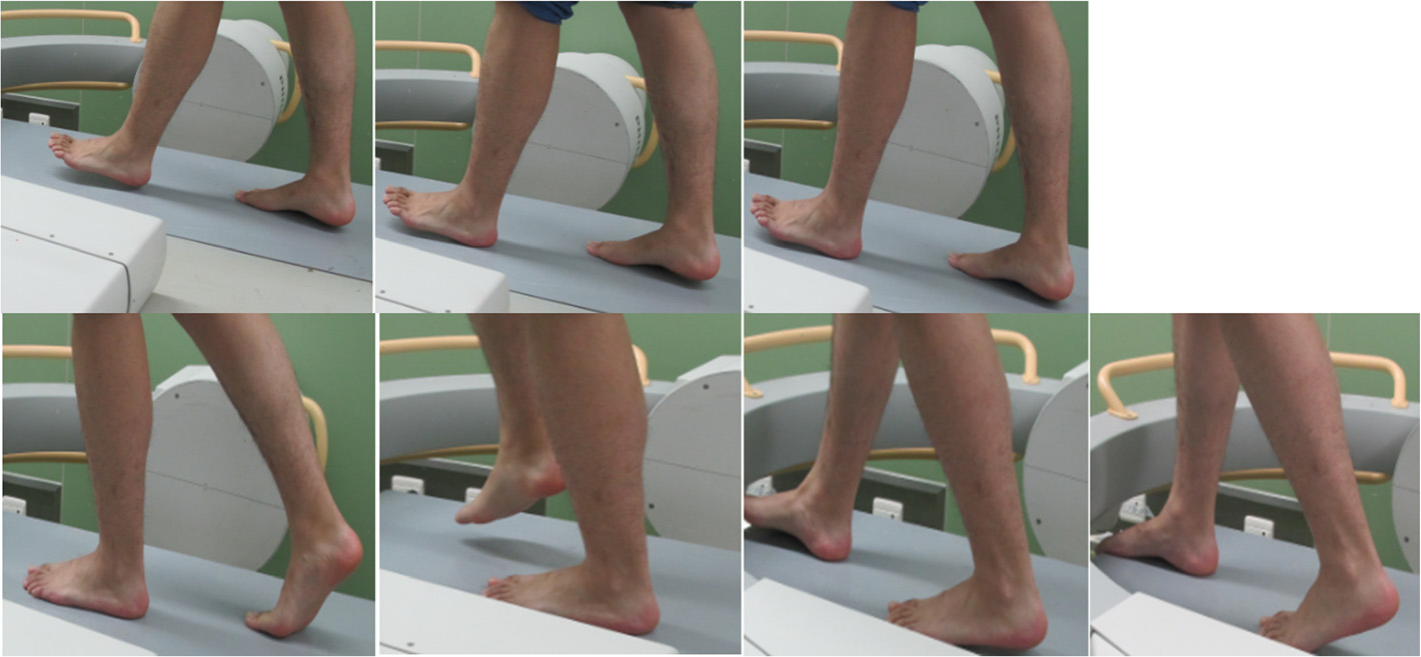
The spatial position of the fibula relative to the tibia after 3D to 2D registration procedure

The 3D bone models were projected onto the distortion-corrected fluoroscopic images in the 3D/2D registration software (FluoMotion, Innomotion Inc., Shanghai). The software created a 3D virtual environment of the experiment in the computer. Each bone segment can be moved and rotated independently at 0.01 mm and 0.01° increments. The software uses canny edge detection algorithms [19] for bone outline detection and optimization algorithm for 3D/2D image-model registration (Fig. 3). The semi-automatic matching algorithm used in the software was previously published [10].

The six DOF spatial positions and orientations of tibia or fibula could be determined then. Bone-to-bone relationship was calculated by the XYZ anatomical coordinate decomposition of the relative transformation of the fibula with respect to the tibia. For each DOF, the mean range of motion (ROM) of the 16 individuals was defined as (ROM_1_ + ROM_2_ + … + ROM_16_)/16; the joint average position at each of the seven poses was calculated as (position_1_ + position_2_ + … + position_16_)/16. The joint motion from pose 1 to pose 4 on each DOF was determined as the difference of joint average positions between the two poses (joint average position of pose 4 and joint average position of pose 1), and in the same way for poses 4 to 7 (joint average position of pose 7 and joint average position of pose 4). Additionally, at each pose, since the joint position values could be either positive or negative for different individuals, the differences between maximal and minimal joint average position values would be smaller than the mean ROM based on the above calculation methods.

## Results

**Fig. 4:**
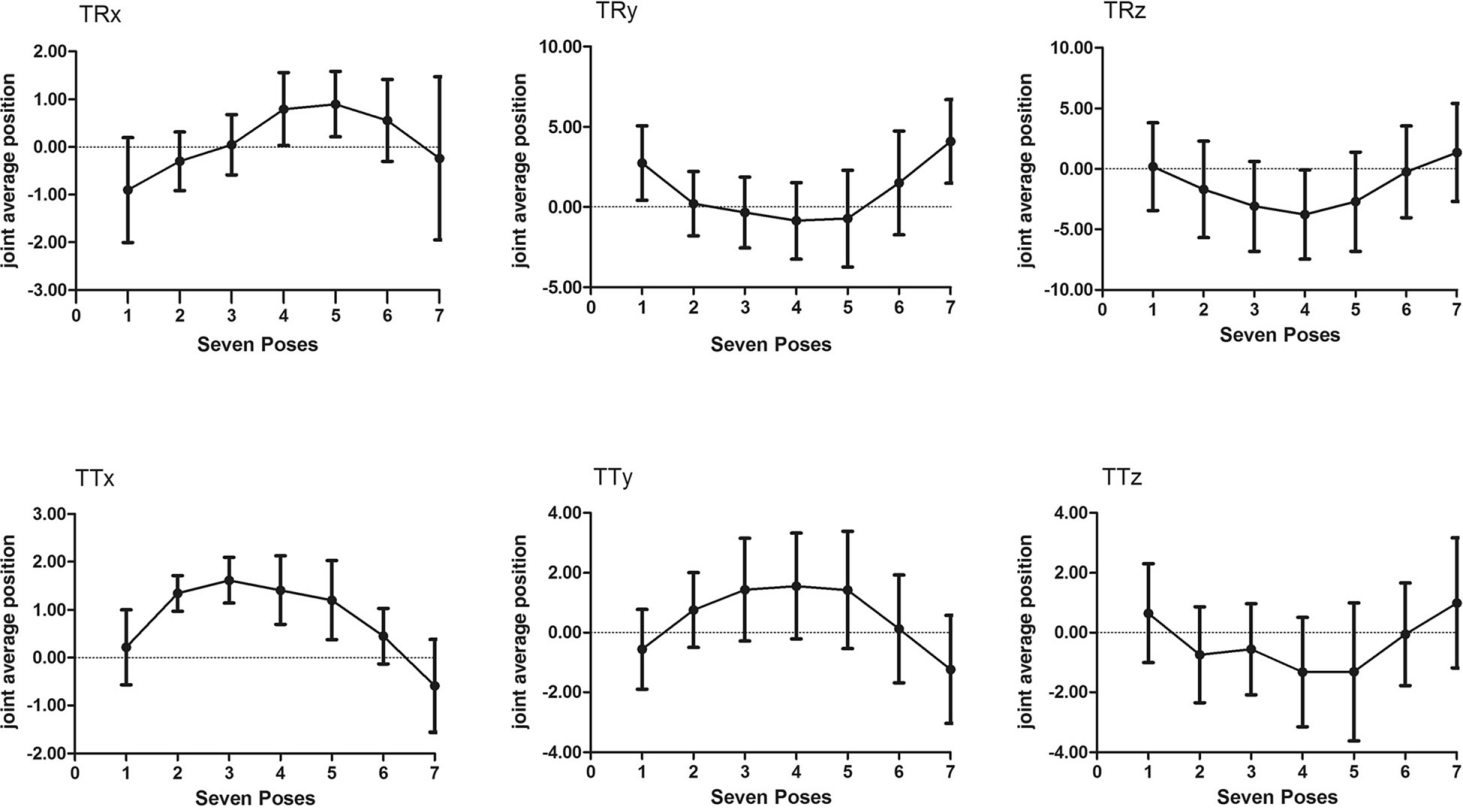
The mean six joint average position in vivo kinematic patterns of the syndesmosis during stance phase (TRx: dorsiflexion(+)/plantarflexion(−); TRy: inversion(+)/eversion(−); TRz: internal(+)/external(−) rotation; TTx: lateral(+)/medial(−) translation; TTy: anterior(+)/posterior(−) translation; TTz: superior(+)/inferior(−) translation)

The mean ROMs of the syndesmosis during stance phase are reported in Table 1, while the joint average positions of the syndesmosis for each DOF are summarized in Table 2. The joint kinematics on each DOF is demonstrated in Fig. 4.

**Table 1:** Mean ROMs of the syndesmosis during the stance phase

Motion	ROM
Dorsi(+)/plantarflexion(−)	2.98 ± 1.10°
Inversion(+)/eversion(−)	5.94 ± 1.52°
Internal(+)/external rotation(−)	5.99 ± 2.00°
Lateral(+)/medialtranslation(−)	2.63 ± 1.05 mm
Anterior(+)/posteriortranslation(−)	3.86 ± 1.65 mm
Superior(+)/inferiortranslation(−)	4.12 ± 1.53 mm

**Table 2:** Joint average positions of syndesmosis on each DOF at the seven poses of stance phase

Lateral(+)/medial(−) translation	Average joint motion
Pose 1	0.22 ± 0.78
Pose 2	1.34 ± 0.37
Pose 3	1.62 ± 0.48
Pose 4	1.41 ± 0.72
Pose 5	1.20 ± 0.83
Pose 6	0.45 ± 0.58
Pose 7	−0.59 ± 0.97
Anterior(+)/posterior(−) translation	Average joint motion
Pose 1	−0.56 ± 1.34
Pose 2	0.76 ± 1.25
Pose 3	1.43 ± 1.72
Pose 4	1.56 ± 1.76
Pose 5	1.42 ± 1.96
Pose 6	0.12 ± 1.81
Pose 7	−1.23 ± 1.81
Superior(+)/inferior(−) translation	Average joint motion
Pose 1	0.65 ± 1.65
Pose 2	−0.74 ± 1.61
Pose 3	−0.56 ± 1.53
Pose 4	−1.32 ± 1.83
Pose 5	−1.31 ± 2.31
Pose 6	−0.06 ± 1.72
Pose 7	0.99 ± 2.17
Dorsi(+)/plantar(−)flexion	Average joint motion
Pose 1	−0.90 ± 1.10
Pose 2	−0.30 ± 0.61
Pose 3	0.05 ± 0.63
Pose 4	0.80 ± 0.76
Pose 5	0.90 ± 0.70
Pose 6	0.60 ± 0.86
Pose 7	−0.24 ± 1.71
Inversion(+)/eversion(−)	Average joint motion
Pose 1	2.75 ± 2.33
Pose 2	0.21 ± 2.01
Pose 3	−0.34 ± 2.21
Pose 4	−0.86 ± 2.38
Pose 5	−0.72 ± 3.01
Pose 6	1.51 ± 3.24
Pose 7	
Internal(+)/external(−) rotation	Average joint motion
Pose 1	0.17 ± 3.63
Pose 2	−1.71 ± 3.99
Pose 3	−3.10 ± 3.70
Pose 4	−3.78 ± 3.68
Pose 5	−2.71 ± 4.09
Pose 6	−0.26 ± 3.79
Pose 7	1.35 ± 4.05

### Mean ROM during the stance phase

The distal tibiofibular joint exhibited different ROMs on the rotational directions with 2.98 ± 1.10° on dorsi/plantarflexion, 5.94 ± 1.52° on inversion/eversion, and 5.99 ± 2.00° on internal/external rotation. The translational motions of the syndesmosis along the medial/lateral, anterior/posterior, and superior/inferior directions were 2.63 ± 1.05, 3.86 ± 1.65, and 4.12 ± 1.53 mm, respectively.

### Joint kinematics during the stance phase

#### From heel strike to mid-stance (pose 1 to pose 4)

From pose 1 to pose 4, the differences of joint average positions between the two poses showed that the syndesmosis demonstrated 1.69° of dorsiflexion, 3.61° of eversion, and 3.95° of external rotation. The mean translational motions were 1.19 mm laterally, 2.12 mm anteriorly, and 1.97 mm inferiorly.

#### From mid-stance to toe-off (pose 4 to pose 7)

From pose 4 to pose 7, the differences of joint average positions between the two poses showed that the syndesmosis demonstrated 1.04° of plantarflexion, 4.95° of inversion, and 5.13° of internal rotation. The mean translational motions were 2.00 mm medially, 2.97 mm posteriorly, and 2.31 mm superiorly.

## Discussion

The syndesmosis maintains the integrity between the distal tibia and the fibula, and resists the axial, rotational, and translational forces that attempt to separate these two bones [1]. However, our knowledge of the syndesmosis during normal gait is limited.

In this study, 3D/2D registration technique was used to investigate the in vivo kinematics of the syndesmosis during walking in a non-invasive way. A validation experiment was also conducted to confirm the accuracy of the 3D/2D technique when applied for the syndesmosis to support our findings (see Appendix).

The current study found that the tibiofibular joint demonstrated with 1.69° of dorsiflexion, 3.61° of eversion, and 3.95° of external rotation from heel strike to mid-stance and 1.04° of plantarflexion, 4.95° of inversion, and 5.13° of internal rotation from mid-stance to heel-off. This can reflect the behavior that the syndesmosis dorsiflexed, everted, external rotated from heel strike to mid-stance, and then moved to the reverse direction from mid-stance to toe-off.

Previous kinematic data about the syndesmosis can be classified as in vitro [1, 3, 9] and in vivo [13, 20], and results of the current study were compared with those of the previous in Table 3.

**Table 3:** Rotational ROMs of the syndesmosis during stance phase in different studies

ROM	Condition	Dorsi(+)/plantarflexion (−)	Inversion(+)/eversion(−)	Internal(+)/external rotation(−)
Present study	Normal gait 3D/2D registration	2.98 ± 1.10°	5.94 ± 1.52°	5.99 ± 2.00°
Huber et al. [9]	Cadaver maximal plantar to dorsiflexion	1.18 ± 0.57°	1.18 ± 0.36°	2.78 ± 1.08°
Lundgren et al. [13]	Normal gait intracortical pins	4.78 ± 1.68°	3.38 ± 1.28°	3.58 ± 1.28°
Arndt et al. [12, 20]	Slow running intracortical pins	3.3 ± 2.4°	2.3 ± 0.9°	1.6 ± 0.3°

In vitro cadaveric study conducted by Huber et al. [9] measured the ROMs of the syndesmosis from maximal plantar flexion to maximal dorsiflexion. The rotational motions (SD) are shown to be 1.18° (0.57°) in plantar/dorsiflexion, 1.18° (0.36°) in inversion/eversion, and 2.78° (1.08°) in internal/external rotation.

Lundgren [13] found that the motion between the fibula and the tibia was small and inconsistent between subjects in normal gaits. The mean ROM (SD) between fibula and tibia was 4.78° (1.68°), 3.38° (1.28°), and 3.58° (1.28°) in the plantar/dorsiflexion, inversion/eversion, and internal/external rotation, respectively. They also demonstrated that the rotations that occurred between the fibula and the tibia were lower than any other joint in the foot.

In vivo studies with intraosseous pins conducted by Arndt et al. [12] found 3.3° of plantar/dorsiflexion, 2.3° of inversion/eversion, and 1.6° of internal/external rotation in the distal syndesmosis during slow running. They found that the sagittal rotation was the major motion while in our study, the internal/external rotation played a major role in the stance phase. We believe this was caused by the different experimental conditions, as the subjects in our experiment were walking in a relatively slow speed while their subjects were tested under slow running. Also, different human species and inter-subject variations may contribute to the difference between the studies.

The kinematic results of the current study were also meaningful to the clinical practice. For instance, we found that the vertical translation and the internal/external rotation were the main motions that occurred in syndesmosis during gait, and such results may again make us consider the current controversy between new-emerging suture button fixation devices and traditional screw fixation after syndesmotic injuries. The suture button device would allow the occurrence of the main two motions while limiting the separation of the syndesmosis. However, also because of the existence of these main motions, it would be hard for the injured syndesmotic ligament to heal at proper length and position and may still be instable after removal of the internal fixation.

Understanding of the physiologic motion of the syndesmosis during walking is the basis for further researches about more complicated motions, such as some sports. The six DOF kinematic data of the current study added quantitative data to the in vivo database of normals and also would be helpful in future development with regard to the clinical diagnosis, treatment, and evaluation of syndesmotic injuries.

## Conclusions

During the stance phase of normal gait, internal/external rotation and vertical motion play dominant roles in terms of rotation and translation, respectively. From heel strike to mid-stance, the fibula dorsiflexed, everted, external rotated relative to the tibia, and then moved to the reverse direction from mid-stance to toe-off.

## Figures and Tables

**Fig. 5:**
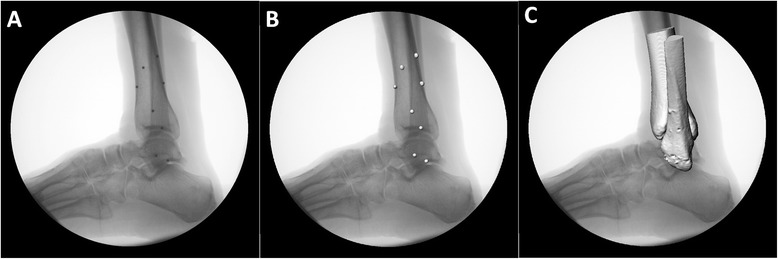
**a** The titanium beads implanted into the tibia and fibula. **b** The RSA method was regarded as the gold standard by tracking the implanted beads. **c** The 3D to 2D registrations of the syndesmosis

## Appendix

It was the first time for the 3D/2D registration technique to be introduced to measure the kinematics of the distal syndesmosis, and it has been reported to have relative larger out-plane errors [10]. We thus additionally conducted a cadaveric validation test (see Appendix) to measure the accuracy and precision of the single plane 3D/2D registration technique when applied to the syndesmosis.

One fresh-frozen cadaveric below knee specimen (age 63 years old; gender female; BMI 23.8) was used in the validation experiment to test the accuracy and precision of 3D/2D registration technique. The specimen was obtained from the voluntary body donation center at Shanghai Medical School, Fudan University. The specimen was preserved at −18 °C and thawed for 12 h at room temperature before the experiment.

The roentogen-sterometric analysis (RSA) method was the gold standard, and the spatial motions of tibia and fibula measured by 3D/2D registration were compared with the RSA results in six DOF. Every four 2.5-mm titanium beads were widely implanted into the tibia and fibula to improve the accuracy of RSA results.

The tibia and fibula was manipulated to simulate three physiological motions including dorsi/plantarflexion, internal/external rotation, and inversion/eversion at maximal range when the lateral X-ray images were captured by fluoroscopy. Five fluoroscopic images were randomly selected from each movement arc, and thus totally, 15 images were used for follow-on validation analyses. The RSA method was applied to determine the six DOF spatial position of the syndesmosis by tracking the four implanted titanium beads with the standard RSA algorithm (Fig. 5).

The 3D/2D registration of the tibia and fibula was repeated ten times on each selected image, and the mean six DOF data were obtained using the procedure described above (“Materials and methods” section). The mean errors of the 3D/2D registration technique, when applied to the syndesmosis at six DOF, could then be calculated by comparing with the RSA method. The mean errors were used to report the accuracy of 3D/2D registration technique (Δ*X*, Δ*Y*, and Δ*Z* were the average translational errors while Δ*α*, Δ*β*, and Δ*γ* were the average rotational errors along the X, Y, and Z axes). The standard deviation of the ten repeated 3D/2D registration results therefore represented the precision or repeatability of this technique.

The accuracy and repeatability of the single plane 3D/2D registration technique to reproduce the syndesmosis are summarized in Table 4.

**Table 4:** Accuracy (average error values) and precision (standard deviations of the automatic matching procedure) error between the steel ball and model coordinates

Error: avg ± std	Translation (mm)			Rotation (deg)		
	∆*x*	∆*y*	∆*z*	∆*α*	∆*β*	∆*γ*
Fibula	–0.64 ± 1.36	–0.22 ± 0.72	–0.26 ± 0.45	0.22 ± 0.45	–0.65 ± 0.63	0.4 ± 1.23
Tibia	0.82 ± 0.9	0.33 ± 0.47	–0.55 ± 0.22	0.19 ± 0.4	0.56 ± 0.4	–0.52 ± 1.04
Tibiofibular joint	–0.86 ± 1.28	0.38 ± 0.72	–0.29 ± 0.86	0.36 ± 0.49	–0.82 ± 1.56	0.73 ± 1.12

Except for the medial/lateral translational direction, the mean out-plane errors are all less than 1° in rotation and the in-plane errors are less than 0.5° in rotation and 0.5 mm in translation. This result substantiates the fact that the single fluoroscopic images could produce satisfactory accuracy in the syndesmosis. The maximal error was detected along medial/lateral translation to be −1.86 ± 1.28°and would effect our kinematic data on this translational direction in certain extent. However, since the main translational motion in the syndesmosis existed in the vertical plane and measured to be much larger than the medial/lateral translation, such out-plane translational error had no impact on our final conclusions. But we still recommended that some cautions should be taken when interpreting medial/lateral translational data.

Our validation results are consistent in accuracy with the published data in previous experiments of the knee and shoulder joints conducted by Zhu et al. [10]. They also found the single plane can achieve in-plane error of less than 0.5 mm in translation and 1° in rotation. A relatively large error was also detected in the out-of-plane translational direction.

In conclusion, the single plane 3D/2D registration can accurately reproduce the in vivo kinematics of the distal tibiofibular joint. Additionally, due to its advantage of simplicity in practical application, we suggested that such technique should be more widely used to resolve some problems in terms of injury diagnosis, treatment, and surgical evaluation of the distal tibiofibular syndesmosis.
